# Combined treatment with emodin and a telomerase inhibitor induces significant telomere damage/dysfunction and cell death

**DOI:** 10.1038/s41419-019-1768-x

**Published:** 2019-07-11

**Authors:** Rui Liu, Jing Liu, Shuqing Wang, Yinsong Wang, Tao Zhang, Yang liu, Xin Geng, Feng Wang

**Affiliations:** 1 Department of Genetics, School of Basic Medical Sciences, Tianjin Medical University, 300070 Tianjin, China; 2 School of Pharmacy, Tianjin Medical University, 300070 Tianjin, China; 3 Tianjin Key Laboratory of Radiation Medicine and Molecular Nuclear Medicine, Institute of Radiation Medicine, Chinese Academy of Medical Sciences and Peking Union Medical College, 300192 Tianjin, China; 4 Department of Biochemistry and Molecular Biology, School of Basic Medical Sciences, Tianjin Medical University, 300070 Tianjin, China

**Keywords:** Drug development, Telomeres

## Abstract

G-quadruplex telomeric secondary structures represent natural replication fork barriers and must be resolved to permit efficient replication. Stabilization of telomeric G4 leads to telomere dysfunctions demonstrated by telomere shortening or damage, resulting in genome instability and apoptosis. Chemical compounds targeting G4 structures have been reported to induce telomere disturbance and tumor suppression. Here, virtual screening was performed in a natural compound library using PyRx to identify novel G4 ligands. Emodin was identified as one of the best candidates, showing a great G4-binding potential. Subsequently, we confirmed that emodin could stabilize G4 structures in vitro and trigger telomere dysfunctions including fragile telomeres, telomere loss, and telomeric DNA damage. However, this telomere disturbance could be rescued by subsequent elevation of telomerase activity; in contrast, when we treated the cells with the telomerase inhibitor BIBR1532 upon emodin treatment, permanent telomere disturbance and obvious growth inhibition of 4T1-cell xenograft tumors were observed in mice. Taken together, our results show for the first time that emodin-induced telomeric DNA damage can upregulate telomerase activity, which may weaken its anticancer effect. The combined use of emodin and the telomerase inhibitor synergistically induced telomere dysfunction and inhibited tumor generation.

## Introduction

Telomeres, specialized protein–DNA structures at the ends of chromosomes that contain runs of guanines, are thought to play an important role in genomic stability. Telomere length can be maintained by the action of telomerase by adding TTAGGG repeats to the chromosome ends^1,2^. Telomerase is a cellular ribonucleoprotein reverse transcriptase that is almost universally detected in immortalized human cell lines and cancer cells^3^. When there is a lack of sufficient levels of telomerase, telomere DNA repeats decrease with cell division, and the telomeric cap is gradually deprotected^2^. Thus, telomerase is required in tumor and immortal cells for an increased replication potential^4^. As potential cancer therapy agents, telomerase inhibitors have been reported to lead to cell death through apoptosis in cancer cells^5–7^. However, following the blockade of telomerase, a population of cells becomes resistant and regains even better viability, which might be due to the reactivation of telomerase, initiation of the alternative telomere maintenance mechanism (ALT) or other related signaling pathways^8^. An additional role of telomerase beyond telomere maintenance has been discovered, and previous studies showed that DNA damage could upregulate telomerase activity, resulting in the addition of de novo telomeric DNA fragments directly to nontelomere breaks^9–12^. A more recent study indicated that telomerase assembly and recruitment are also regulated by ATM and ATR, which are two key proteins that play essential roles in the DNA damage response^13^.

Due to their G-rich and repetitive nature, telomeres can adopt secondary structures known as G4s^14^. G4s consist of stacks of G-quartets formed by four guanines via Hoogsteen base pairing. The stabilization of G4s at telomeres impacts telomere association, recombination, and replication, leading to telomere dysfunction, observed as incomplete end-replication, abnormal telomere DNA breakage, loss of telomere capping or critical telomere shortening^15–17^. When telomeres become dysfunctional, DNA damage response factors such as γ-H2AX, 53BP1, and the MNR complex are recruited to form telomere dysfunction-induced foci (TIFs)^18–20^. The improper repair of disturbed telomeres could result in genomic instability and cellular apoptosis. In addition, G4s formed at the ends of telomeres could impede telomerase recognition and inhibit telomere elongation, leading to telomere shortening^21,22^. Thus, the use of G4 ligands that stabilize the G4 structure tends to cause stalling the replication fork and induction of telomere DNA damage. Therefore, the stabilization of G4 at telomeres has been considered a promising strategy in the field of anticancer therapy to kill highly proliferating cells^23^.

A virtual screening assay was used here to identify potential G4 ligands from the database of natural compounds originating from traditional Chinese medicine (TCM). Emodin was identified as one of the best potential substrates, showing a good docking score with the structure. Emodin (1,3,8-trihydroxy-6-methyl-anthraquinone) has received much research interest due to its anticancer effects^24,25^. Emodin has been reported to modulate cellular chronic inflammation and cancer cell proliferation via inhibition of nuclear factor-κB (NF-κB). On the other hand, previous studies suggested that emodin causes DNA double-strand breaks (DSBs), likely via inhibition of topoisomerase (topo) II activity through stabilization of topo II-DNA complexes^26^. Furthermore, emodin has been shown to promote intracellular reactive oxygen species (ROS) generation^27–29^, which may trigger DNA damage and cell cycle arrest^30,31^. However, emodin has not been widely used in clinical trials as an oncotherapeutic agent due to its poor oral bioavailability and cytotoxicity against normal cells^24^.

Here, we found that emodin treatment arrested cancer cell proliferation by activating apoptosis and the senescence pathway. However, the inhibition of proliferation did not last for a long period and recovered later a few days after treatment. Subsequent analyses showed that emodin could stabilize the G4 structure and that the stabilization of G4 led to significantly increased telomere deficiencies, including multiple telomere signals (MTSs), single free ends (SFEs) and TIFs. Interestingly, we observed that the dysfunctional telomeres could be restored by increased telomerase activity, which might be triggered by emodin-induced DNA damage. However, the application of the telomerase inhibitor BIBR1532 upon emodin treatment obstructed the rescue of the deficiency and significantly inhibited tumor growth in a mouse model. Taken together, our data indicated that the combination of emodin and telomerase inhibitors might serve as a potential oncotherapeutic agent for telomerase-positive cancers by causing persistent telomere damage.

## Materials and methods

### Molecular docking

The parallel (1KF1), antiparallel (143D)^32^, and hybridized (2JPZ) G4 structures were used as receptors for virtual screening studies. The most comprehensive and largest non-commercial TCM (Traditional Chinese Medicine Database)^33^ available is downloaded from ZINC database (10.1021/ci3001277). The parameter files for the two G4 structures and the natural compound database were prepared by PyRx, and the virtual screening was done by AutoDock Vina^34^ as a docking engine. A grid box of 100, 100, and 100 in *x*, *y*, and *z* directions were set up with a grid spacing of 0.375, surrounding the active pocket of the G4. The natural compounds were subsequently docked to the two G4 structures via AutoDock Vina respectively^35^.

### Cell culture and compounds

Human cervix epithelioid line HeLa1.2.11 cells were cultured in RPMI 1640 (purchased from Biological Industries), Human colon cancer HCT116 cells, Human breast cancer MCF-7 cells and Mouse breast cancer 4T1 cells were cultured in DMEM (purchased from Biological Industries), all of them supplemented with 10% fetal bovine serum (purchased from Gibco) and 1% penicillin-streptomycin and maintained at 37 °C in a humidified incubator with 5% CO_2_. Fresh medium was added after every 2 d.

Emodin, 6-methyl-1,3,8-trihydroxyanthraquinone, was purchased from Sigma. Emodin was dissolved in DMSO (Sigma) and stored at 4 °C. Lower dosage of emodin for long-term treatment related experiments and higher dosage for most of the other experiments.

### Antibodies

Anti-γ-H_2_AX mouse antibody (1:2000; Millipore, Temecula, CA, clone JBW301). 53BP1 rabbit antibody (1:5000; NOVUSBIO, NB100-304). The secondary antibodies used were conjugated with Alexa Fluor 555 goat anti-mouse (1:2000; Invitrogen). BG4: provided by Dr. Shankar Balasubramanian (Univerisity of Cambridge, UK) for BG4 antibody (plasmid). Flag-tag (MA4) Mouse Monoclonal Antibody (1:1000) and Alexa Fluor 555 Goat Anti-Mouse IgG (H + L) Antibody (1:2000).

### Cell proliferation assay

Cells were seeded into 10 cm plate for 30 days (Fig. 2a) or 10 days (Fig. 6a). Cell passage was performed every 2 days and counted. Different concentrations of drugs were added into plate. The cell growth curve was obtained directly by cell counting. Cells were seeded into 96-well plates at 10^4^ per well (Fig. S1A & Fig. S5A–D). After overnight culturing, different concentrations of drugs were added into wells. Cell growth inhibition was measured by CCK-8 (Dojindo Molecular Technologies, Rockville, MD, USA) assays 48 h later. The cytotoxicity was presented as IC 50, which is the toxin concentration that reduced cell viability by 50% compared with the cells that were not treated with the toxin.

### Cell cycle analysis

HeLa cells were grown as described above. After 48 h exposure to emodin, the cells were detached by trypsinization, washed with PBS, then centrifugated 2 times at 1100 rpm for 4 min, and fixed in 70% ethanol at 4 °C overnight. The cells were then washed with PBS and stained with 50 μg/ml PI for 30 min. PI fluorescence was measured using a flow cytometer (BD FACSCalibur^TM^ Flow Cytometer, E34297502922).

### RNA Isolation and RT-PCR for hTERT

RNA extracted using the the Eastep^®^ Super Total RNA Extraction Kit (Promega) according to the manufacturer’s instructions. In total, 1 μg of total RNA was reverse-transcribed using the HiScript^®^ II Q Select RT SuperMix Specific primers were designed to the boundary spanning regions of the α and β subunits of hTERT, because this has been shown to be an effective way to detect splice variants. Primers hTERT α subunits 2172 (5′-TGTACTTTGTCAAGGTGGATGTG-3′) and β subunits 2350 (5′-GTACGGCTGGAGGTCTGTCAA-3′)^36^.

### β-galactosidase staining

The experiment was performed using a Senescence β-Galactosidase Staining Kit (Beyotime) following the instructions of the manufacturer. Cells were washed once in PBS, fixed for 15 min at room temperature in 3% formaldehyde, and washed three times with PBS again. Then, cells were incubated overnight at 37 °C overnight in a dry incubator in the absence of CO_2_ before they were examined under a microscope to assess blue color development. At least 300 cells were counted in randomly chosen fields.

### TUNEL assay

Cells were grown on glass coverslips, fixed with 4% paraformaldehyde solution and permeabilized with Triton X-100 (1%). For TUNEL assay, DNA strand breaks were detected with a TUNEL assay kit (KeyGEN BioTECH, KGA7051) according to the manufacturer’s instructions. Nuclei were stained with DAPI. Fluorescent images of at least 20 metaphases of two different experiments were obtained by fluorescence microscope (Nikon Eclipse Ti, Japan). For each group, the number of apoptotic cells and the total number of cells in ten random fields (magnification, × 20) were photographed and counted.

### Telomere DNA FISH

Metaphases chromosomes were prepared as previously described^37^. Briefly, actively dividing cells were kept in 0.1 mM colcemid for 2 hours then centrifuged at 1000 rpm for 5 min. The cells were washed in PBS, swelled with 0.075 M KCl at 37 °C for 20 min and fixed in methanol: acetic acid (3:1). Cell suspensions were then dropped on chilled slides and dried at 85 °C for 1–2 min. Cells fixed on glass slides were incubated with FITC-conjugated peptide nucleic acid (PNA) telomeric oligonucleotide (CCCTAA)_3_ probe at 37 °C for 2 h. Fluorescent images of at least 20 metaphases of two different experiments were obtained by fluorescence microscope (Nikon Eclipse Ti, Japan).

### Telomere repeats amplification protocol for telomerase activity

Telomerase activity of HeLa cell was assayed with telomeric repeat amplification protocol (TRAP) performed as previously described^38^. Briefly, after washing with PBS, 10^6^ cells were re-suspended in 500 µL 1x CHAPS lysis buffer, incubated on ice for 30 min, and then centrifuged at 13,000 g at 4 °C for 30 min. In all, 1 µL (2000 cells) was used for the PCR reaction, primers were TS (5′-AATCCGTCGAGCAGAGTT-3′), ACX (5′-GCGCGG[CTTACC]3CTAACC-3′), NT (5′-ATCGCTTCTCGGCCTTTT-3′) and TSNT (5′-ATTCCGTCGAGCAGAGTTAAAGGCCGAGAAGCGAT-3′). Amplified products were separated on 12% nondenaturing polyacrylamide gels and then stained with SYBR Green I (FMC) for 30 min, visualized by UVP imaging system. Fluorescence density was quantified by Image J.

### Animals and tumor model establishment

SPF Balb/c mice (aging 4–5 weeks, weighing 14–19 g, female) were bought from Beijing Vital River Laboratories (China). They were housed in an air-conditioned room at 23 ± 2 °C with free access to food and water and maintained on a 12-h light–dark cycles. Mice were allowed to acclimate to these conditions for at least 7 days before starting experiments. All animal treatments were strictly in accordance with the International Ethics Guidelines and the National Institutes of Health Guidelines Concerning the Care and Use of Laboratory Animals. 4T1 breast tumor spontaneously developed in Balb/c mice. So 4T1 cells (estrogen receptor (ER)/progesterone receptor (PR)-negative) suspensions were prepared with PBS to adjust to the concentration of 5 × 10^6^ cells/ml, then 0.1 ml solution were injected on the groin of each mouse to finish the subcutaneous vaccinations. About 5 days later, rice-sized lumps could be seen on the groin of each mouse, the diameter of which grew to 0.5–0.6 cm, indicating that tumor models were successfully established and subsequent experiments would be able to carry out.

The tumor volumes were measured every 2 days in two dimensions with vernier calipers. The tumor volumes were calculated using the following formula: Length × width^2^ × 0.5. Simultaneously, the body weight of mice were also measured every 2 days.

### Statistics

SPSS 16.0 software was used for statistical analysis. Student’s t test was applied for comparison of the means of two groups. For all of the value differences, *P*  < 0.05 was considered statistically significant.

## Results

### Emodin is screened as a potential G4 ligand that could stabilize the formation of G4s in vitro

**Fig. 1:**
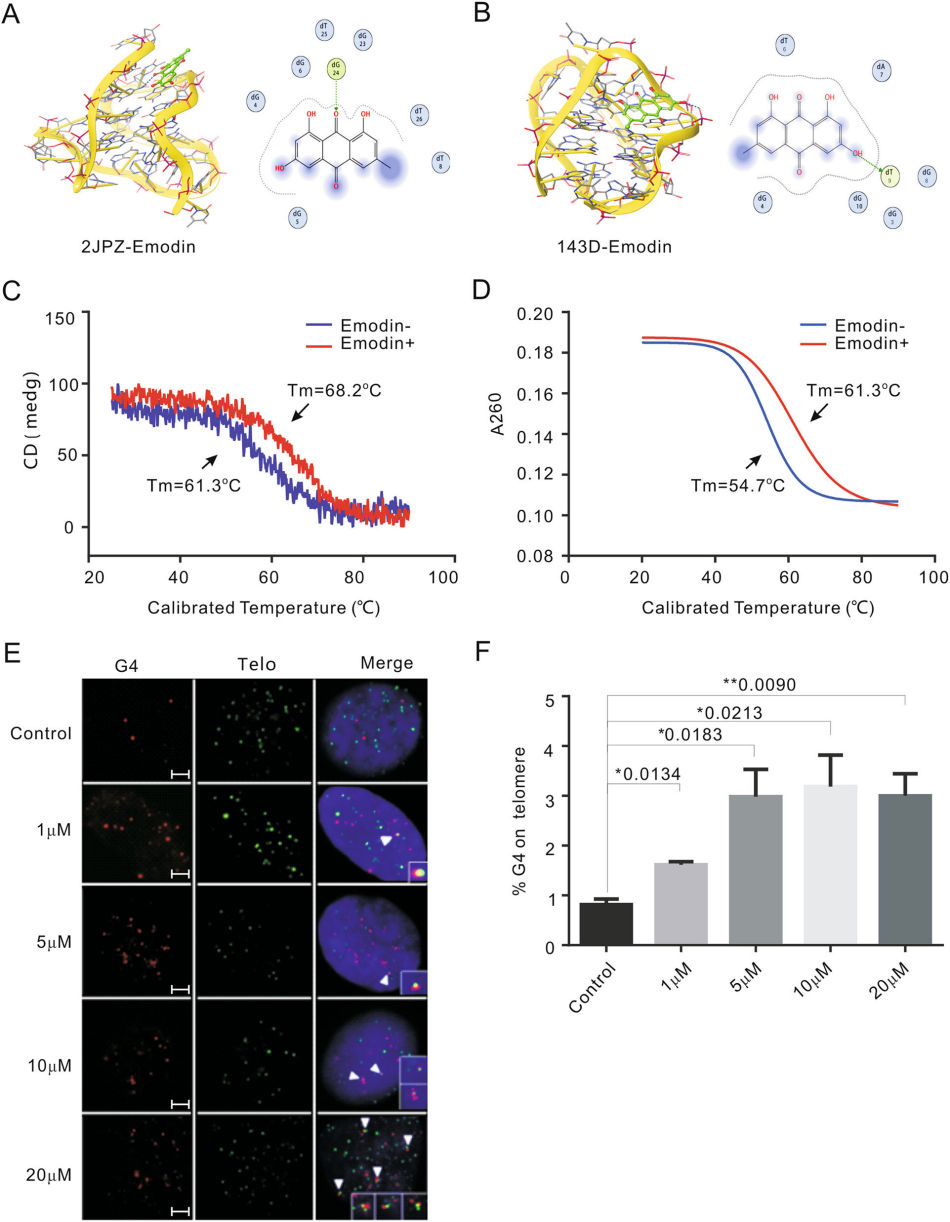
Emodin stablizes the formation of G4 in vitro. **a**, **b** A molecular docking complex of emodin with hybridized and antiparallel G4. **c** Circular dichroism melting spectrum reflected the G4 stability with or without emodin treatment by the melting temperature (*T*_*m*_). **d** The UV melting reflected the G4 stability with or without emodin treatment by the melting temperature (*T*_*m*_). **e** Representative images showing the co-localization of G4 and telomere for the control (DMSO) and emodin-treated HeLa cells (1, 5, 10, and 20 μM for 48 h). The G4 was detected by G4 antibody BG4 (red) and telomere was detected by telomeric FITC-conjugated PNA probes (green). Nuclei were stained with DAPI (blue). White arrowheads in merged images indicate co-localized of G4 and telomere. Scale bars are 2 μm. **f** Graph shows the percentage of G4 on telomere upon emodin treatment. Data are shown as mean ± SD. More than 100 nuclei were analyzed in three independent experiments

Given that G4 ligands may exhibit an antitumor function through disturbance of telomere replication and telomerase inhibition, we conducted virtual screening for potential G4 ligands using PyRx in ZINC, which presents the largest and most comprehensive TCM database generated to date. Considering that parallel, antiparallel and hybridized G4 structures co-exist in cells, all three structures were used as receptors in the PyRx virtual screen. However, no ligands were observed for parallel G4 due to the lack of a pocket site on its surface. And six candidates were obtained as G4 ligands for two other G4 structures based on the docking score. The structures and docking scores are listed in Table 1. Interestingly, all of these compounds showed similar anthraquinone-derived structures, and emodin presented the greatest affinity for both structures (Table 1). The potential binding sites and hydrogen bonds are labeled in Fig. 1a, b. Emodin docked at the binding site of 143D in a larger groove than in 2JPZ, formed by the base pairs 3, 4, and 6–10, and the hydroxyl of emodin donates its H atom to the phosphoric acid oxygen atom of DT9, forming an H-bond (Fig. 1b).

**Table 1:**
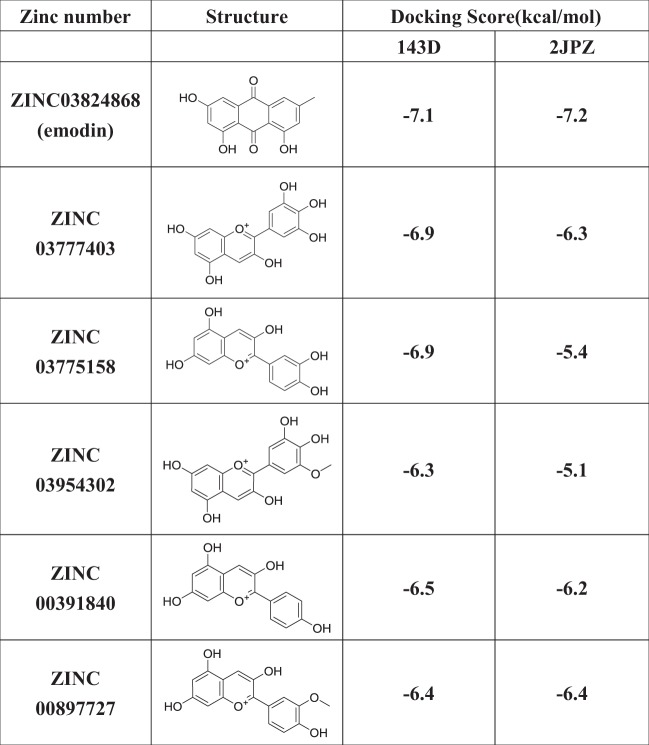
The ZINC numbers and structures of the screened six compounds and their docking scores for 143D and 2JPZ

To further investigate the stabilizing effects of emodin on G4s, a circular dichroism melting spectrum and a UV melting experiment were performed in 150 mM K^+^ and 150 mM Na^+^ respectively, with a gradually increasing temperature. The CD spectrum of 5 μM (TTAGGG)_4_TTA (Tel) DNA was monitored at 295 nm, and we obtained a positive peak for the hybrid G4 structure. G4 stability under different treatments was reflected by the melting temperature (*T*_*m*_). We observed that *T*_*m*_ increased approximately seven degrees from 61.3 °C to 68.2 °C in the presence of emodin (Fig. 1c). The UV melting experiment also revealed that *T*_*m*_ increased approximately seven degrees, from 54.7 °C in the presence of emodin to 61.3 °C when antiparallel G4 was formed in Na^+^ buffer (Fig. 1d), suggesting a significant delay of G4 disruption in vitro upon emodin exposure.

The formation of the G4 structure in the telomere region leads to telomere replication fork stalling and telomere instability^39^. We suspected that emodin might disturb replication through G4 structure stabilization in cells. To verify the capacity of emodin in facilitating the formation of the G4 structure at the telomeric region, an immune-staining assay was performed using a G4 structure-specific antibody (BG4) and a telomeric FITC-conjugated PNA probe. Our data revealed that the percentage of G4s at telomeres was obviously increased upon emodin treatment compared to the control group, suggesting that emodin could induce or stabilize G4 formation (Fig. 1e, f).

### Emodin inhibits cell growth and leads to DNA damage

**Fig. 2:**
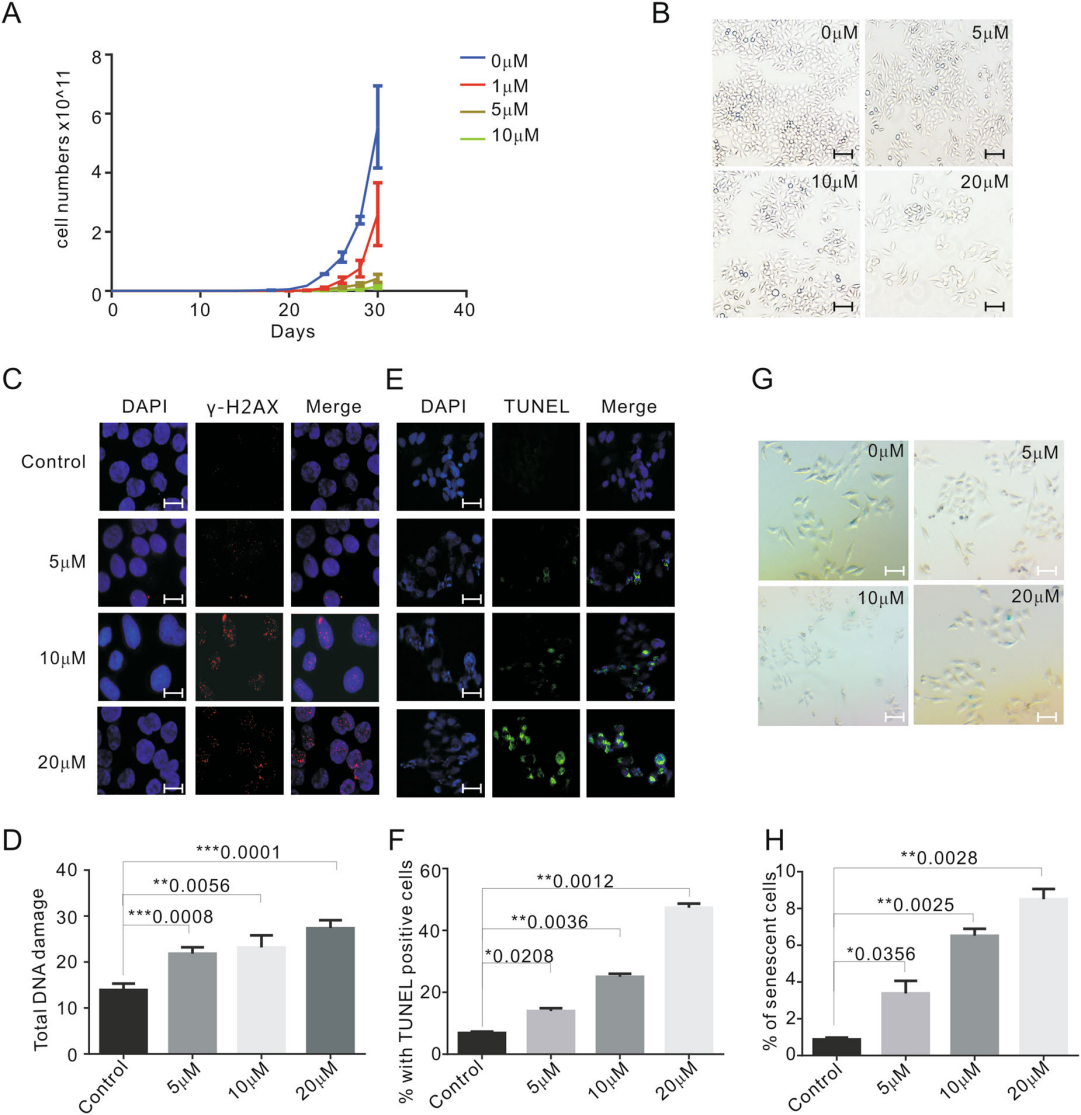
Emodin inhibits cell growth and leads to DNA damage in HeLa cells. **a** Growth curve of HeLa cells treated with DMSO and 1, 5, and 10 μM emodin for 30 days. **b** Morphology of HeLa cells treated with various concentrations of emodin for 48 h. Scale bars are 50 μm. **c** Representative images showing the detection of γ-H_2_AX for the control (DMSO) and emodin-treated HeLa cells (5, 10, and 20 μM for 48 h). γ-H_2_AX (red), nuclei (blue). Scale bars are 10 μm. **d** The graph of the number of DNA-damage foci for **c**. **e** Representative images showing apoptotic cells detected by TUNEL. TUNEL positive cells labeled as green. Cells were treated with DMSO, 5, 10, and 20 μM emodin for 48 h. Scale bars are 20 μm. **f** The graph of **e**. **g** Cells were treated with DMSO and 5, 10, and 20 μM emodin in for 48 h, then sensecense cells were detected by SA-β-gal staining. SA-β-gal-positive cells were stained in blue. Scale bars are 20 μm. **h** The percentage of senescent cells with different treatment. Data are shown as mean ± SD. *n* = 3

It has been reported that emodin showed anticancer potential. Here, to further investigate the cancer cell proliferation inhibition effect of emodin, HeLa and HCT116 cells were treated with the indicated amount of emodin for over 30 population doublings (PDs) or 10 PDs. Proliferation was significantly inhibited upon emodin treatment in a dose-dependent manner (Fig. 2a, b and Fig. S1A). Cellular apoptosis and senescence are the major contributors to cell proliferation blocking. Thus, apoptosis and senescence were detected with or without emodin treatment. The percentage of apoptotic cells was determined by TUNEL assay (Fig. 2e and Fig. S1D). As suspected, the number of apoptotic cells increased upon emodin exposure in both cell lines (Fig. 2f and Fig. S1E). Additionally, β-galactosidase staining was used to distinguish senescent cells (Fig. 2g and Fig. S1F). We observed significantly increased cellular senescence in addition to apoptosis in both cell lines after emodin treatment (Fig. 2h and Fig. S1G). DNA damage is a key trigger for apoptosis and senescence. To further determine the possibility of DNA damage upon emodin treatment, γ-H_2_AX or 53BP1 foci were evaluated. Our results revealed that after 48 h of emodin treatment, the frequencies of γ-H_2_AX foci and 53BP1 foci were increased at least two-folds compared to the DMSO control (Fig. 2c, d and Fig. S1B, C). Taken together, these results indicate that emodin exposure causes global genomic DNA damage, which might be the main cause of the activation of apoptosis and senescence.

The treatment with various G4-ligands has been reported to stall telomere replication forks and lead to telomeric DNA-specific damage^40^. Additionally, telomere damage or telomere replication deficiency promotes cell cycle progression arrest and cell growth inhibition resulting from the activation of the ATR/Chk1 pathway^41^. Thus, to investigate the role of emodin in cell cycle regulation, a flow cytometric analysis was performed 48 h after emodin exposure. The current data (Fig. S1H) showed that similar to other DNA damage reagents or other G4 stabilizers, emodin treatment triggered S-phase cell cycle arrest. Taken together, the above data indicate that emodin treatment leads to telomere damage or telomere replication dysfunction, which then promotes cell cycle arrest, initiates cellular apoptosis, and ultimately inhibits proliferation.

### Emodin induces telomere-specific DNA damage and dysfunction

**Fig. 3:**
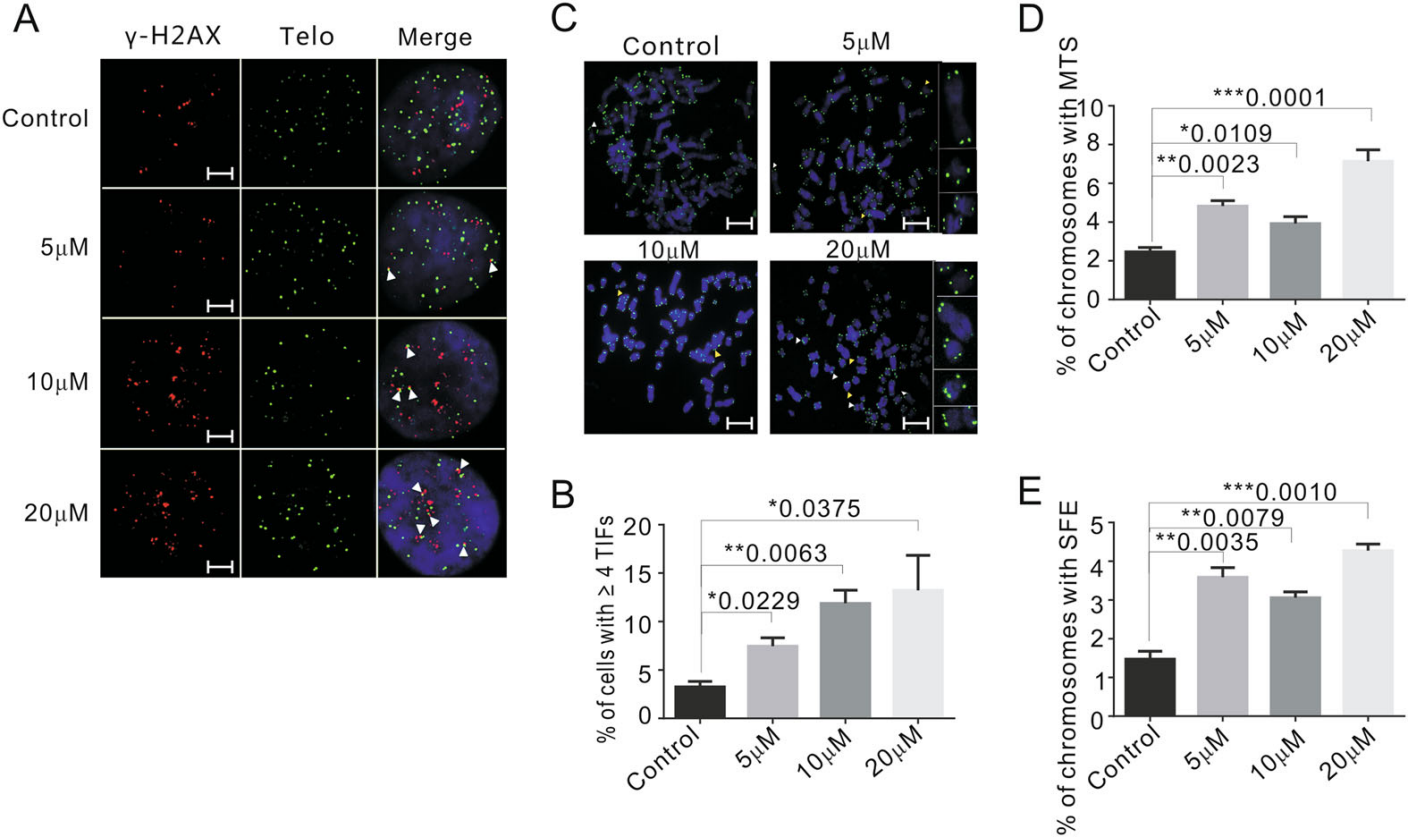
Emodin induces telomere-specific DNA damage and dysfunction. **a** Immunolocalization of γ-H_2_AX (red) and FISH of telomere (green) in HeLa cells grown with or without emodin for 48 h. White arrows indicate TIFs (sites of γ-H_2_AX with telomeres). Scale bars are 2 μm. **b** The percentage of cells with ≥4 TIFs was determined for at least 50 cells in each experiment. **c** Telomere FISH with HeLa cells treated with indicate amount of emodin for 48 h. White arrows indicate MTS and yellow arrows indicate SFE. Scale bars are 5 μm. **d**, **e** Percentage of chromosomes with MTS and SFE. More than 1000 chromosomes were analyzed in each experiment. Data are shown as mean ± SD. *n* = 3

Given that emodin can interact with and stabilize G4 structures, we expected that treatment with emodin might lead to telomere-specific disturbances. Therefore, telomere dysfunction-induced foci (TIFs) were used to evaluate the effect of emodin on telomere integrity. The co-localized telomeric γ-H_2_AX signals were used to demonstrate telomere-specific DNA damage, and cells with more than 4 co-localized signals were counted as TIF-positive cells (Fig. 3a). We observed that 48 h of treatment with 20 μM emodin-induced a significant increase in TIF-positive cells, with the percentage of TIF-positive HeLa cells being increased from 3.2% (DMSO-treated control cells) to 13.9% (20 μM emodin-treated cells; Fig. 3b). Moreover, additional telomere damage was observed in the presence of higher emodin concentrations. Similar phenotypes were observed in HCT116 cells (Fig. S2A, B), which indicated that emodin could lead to telomere-specific DNA damage.

Two other hallmarks of disturbed telomeres are fragile telomeres, in which individual chromatids exhibit multiple telomere FISH signals (MTSs), and signal-free ends (SFEs), in which telomeres lack detectable FISH signals^37,42^. Thus, MTSs and SFEs were detected via telomere FISH in metaphase spreads. Cells were also treated with 5, 10, 20, and 40 μM emodin for 48 h (Fig. 3c and Fig. S2C). The presented data revealed that the number of chromosomes that either exhibited multiple telomeres or lack telomere signals were increased by 48 h of emodin treatment (Fig. 3d, e and Fig. S2D, E). Therefore, we conclude that short-term exposure to emodin caused significant telomere abnormalities, which could reflect replicative stress (MTSs) and breakage at the ends of the chromosomes (SFEs)^43^.

### Emodin induces restorable telomere dysfunction

**Fig. 4:**
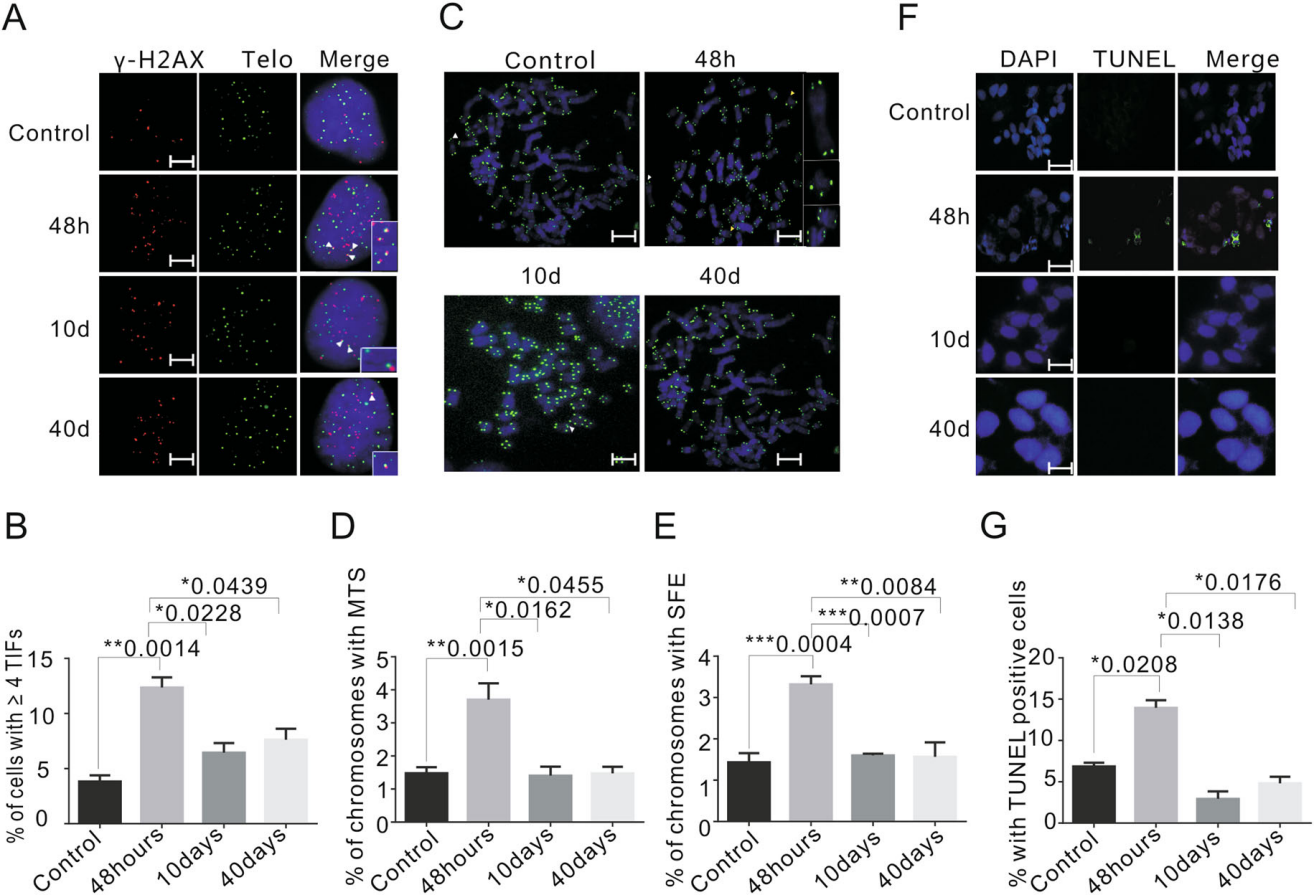
HeLa cells can resist emodin treatment. **a** Representative images showing the TIFs in control and 5 μM emodin-treated HeLa cells (48 h, 10 and 40 days). γ-H_2_AX (red), telomere (green). Nuclei were stained with DAPI (blue). White arrow heads indicate the TIFs. Scale bars are 2 μm. **b** The graph showing the percentage of TIFs with different treatment. **c** Representative images of metaphase telomere FISH. HeLa cells were treated with emodin in 5 μM for 48 h, 10 and 40 days. White arrows indicate MTS and yellow arrows indicate SFE. Scale bars are 5 μm. **d**, **e** Percentage of chromosomes with MTS and SFE for different treatment. **f** Representative images of TUNEL-positive percentage of apoptosis cells were detected by TUNEL with indicated condition. Scale bars are 20 μm. **g** Graph showing the percentage of TUNEL-positive cells after emodin treatment. Data are shown as mean ± SD. *n* = 3

Interestingly, we found that the emodin-induced TIFs and telomere abnormalities did not last for a long period and could be rescued with time. When HeLa cells were cultured in 5 μM emodin, the percentage of TIF-positive cells reached the maximum level (13.5%) on day two (48 h), then gradually decreased and returned to the lowest level (6.9%) on day 10 (Fig. 4a, b). Similarly, the frequency of MTSs and SFEs was increased in the first 2 days after emodin treatment, after which it decreased and remained at a low level (Fig. 4c–e). Furthermore, the number of apoptotic cells determined by TUNEL assays showed the same trend (Fig. 4f, g), suggesting that drug resistance was induced by long-term emodin treatment. The rescue of the telomere dysfunction and apoptosis with time was also observed in other cell lines including HCT116 cells (Fig. S3A–G) and MCF-7 cells (Fig. S3H, I). Interestingly, we found that the number of senescent cells did not recover with time (Fig. S3J, K), which might explain why cell growth could not be rescued (Fig. 2a). Taken together, our data indicated that the telomere defects and other general defects induced by emodin treatment with the exception of cell senescence were repairable by the cells over time.

### Telomerase activity is increased upon emodin treatment

**Fig. 5:**
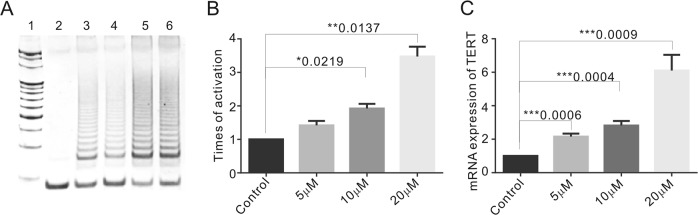
Telomerase activity was elevated in the first few days upon emodin treatment and recovered late on. **a** Telomerase activity was determined by TRAP assay. Lane 1, DNA molecular weight marker; Lane 2, negative control; Lane 3, HeLa cell treated with DMSO, Lane 4–6, HeLa cells treated with 5, 10, and 20 μM emodin for 48 h. **b** Graph showing the telomerase activity for the emodin-treated HeLa. **c** Relative expression of hTERT mRNA in HeLa cells treated in 5, 10, and 20 μM for 48 h by RT-PCR. Data are shown as mean ± SD. *n* = 3

It has been reported that cellular telomere damage can increase telomerase activity in both tumor^10,11,44^. Telomerase can add de novo telomeric DNA at damaged sites for chromosome healing purposes^31^. Moreover, DNA damage response factors including ATM, ATR and Rad9/Rad1/Hus1 have been reported to interact with telomerase and play important roles in telomerase activity regulation^13,45,46^. Thus, we suspected that emodin-induced telomere damage triggers telomerase activity, which then rescues the telomere defects. To further investigate this possibility, a telomere repeat amplification protocol (TRAP) assay was used to detect telomerase activity, and the results revealed that the telomerase activity induced upon emodin exposure was increased by 1.12 ± 0.19-fold (5 μM), 1.78 ± 0.16-fold (10 μM), or 3.47 ± 0.41-fold (20 μM) compared to the DMSO-treated control (Fig. 5a, b). In agreement with the previous hypothesis, our data showed that emodin treatment might lead to upregulation of telomerase activity in response to telomere damage.

To better understand the mechanism of telomerase induction, telomerase reverse transcriptase component mRNA expression was determined by real-time PCR in HeLa and MCF-7 cells. After 48 h of 5, 10, or 20 μM emodin treatment, TERT mRNA transcription was significantly upregulated (Fig. 5c). However, TERT mRNA transcription decreased with the extension of 5 μM emodin treatment (Fig. S4A, B), which might correspond to the decreased level of DNA damage. Taken together, our data suggested that the DNA damage induced by emodin treatment would lead to an increase in telomerase at the transcription level.

### Combined treatment with emodin and a telomerase inhibitor induces permanent DNA damage

**Fig. 6:**
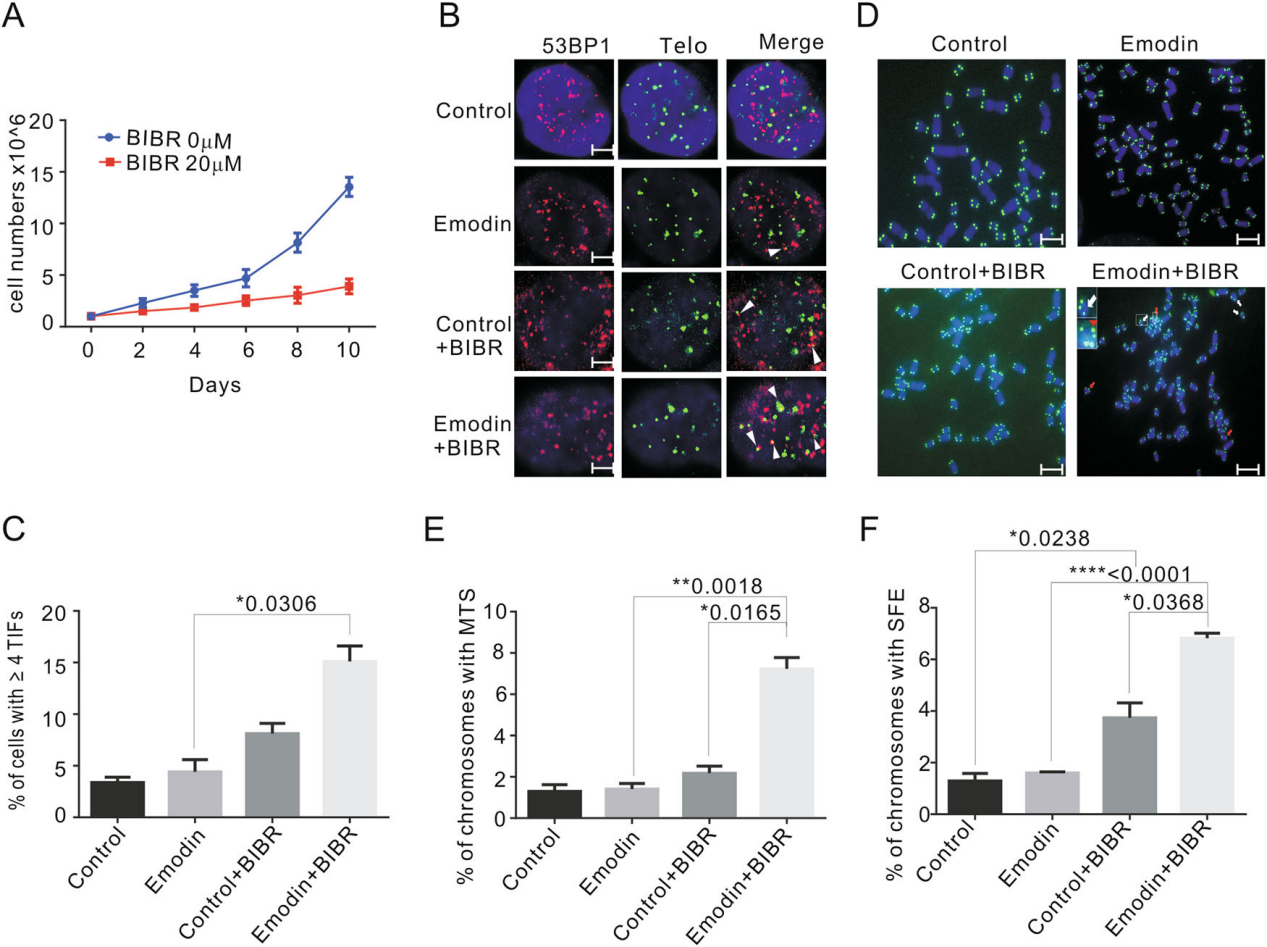
Emodin-induced telomere damage and dysfunction cann’t be rescued when telomerase activity gets inhibited. **a** Growth curve of HeLa cells treated with 5 μM emodin and 0 μM or 20 μM telomerase inhibitor BIBR1532 for 10 days. **b** Immunolocalization of 53BP1 (green) and FISH of telomere (red) in HeLa cells. Control (DMSO for 10 days), emodin group (5 μM for 10 days), BIBR group (DMSO for 48 h, then 20 μM BIBR1532 was added for additional 8 days), emodin and BIBR group (emodin 5 μM for 48 h, then 20 μM BIBR1532 was added for additional 8 days). White arrows indicate TIFs (sites of 53BP1 with telomeres). Scale bars are 2 μm. **c** The percentage of cells with ≥4 TIFs was determined for at least 50 cells in each experiment. **d** Representative images of metaphase telomere FISH. Control (DMSO for 10 days), emodin group (5 μM for 10 days), BIBR group (DMSO for 48 h, then 20 μM BIBR1532 was added for additional 8 days), emodin and BIBR group (emodin 5 μM for 48 h, then 20 μM BIBR1532 was added for additional 8 days). White arrows indicate SFE and red arrows indicate MTS. Scale bars are 5 μm. **e**, **f** Percentage of chromosomes with MTS and SFE with different treatment. Data are shown as mean ± SD. *n* = 3

To investigate the impact of telomerase activity on telomere DNA damage repair, the telomerase inhibitor BIBR1532 was used to inhibit telomerase. HeLa cells were treated with or without 20 μM BIBR1532 in emodin-containing medium for 10 days, and the data showed that cell proliferation was significantly inhibited by the combined treatment (Fig. 6a). The percentage of cell viability was determined with a cell counting kit-8 (CCK-8). We observed that 20 μM BIBR1532 functioned synergistically with different concentrations (5, 10, 20, and 40 μM) of emodin to inhibit cell proliferation in HeLa and MCF-7 cells. (Fig. S5A, B). In contrast, the combined treatment of telomerase-negative cancer cells (U2OS cells, Fig. S5C) and telomerase-null normal fibroblasts (BJ cells, Fig. S5D) did not result in additional inhibition. Additionally, the co-localization of telomeric 53BP1 or γ-H_2_AX signals and telomeric FISH analysis revealed that telomere damage or dysfunction could not be rescued without telomerase in telomerase-positive cancer cell lines, including HeLa (Fig. 6b–f and Fig. S5E, F) and MCF-7 cells (Fig. S5G–I).

**Fig. 7:**
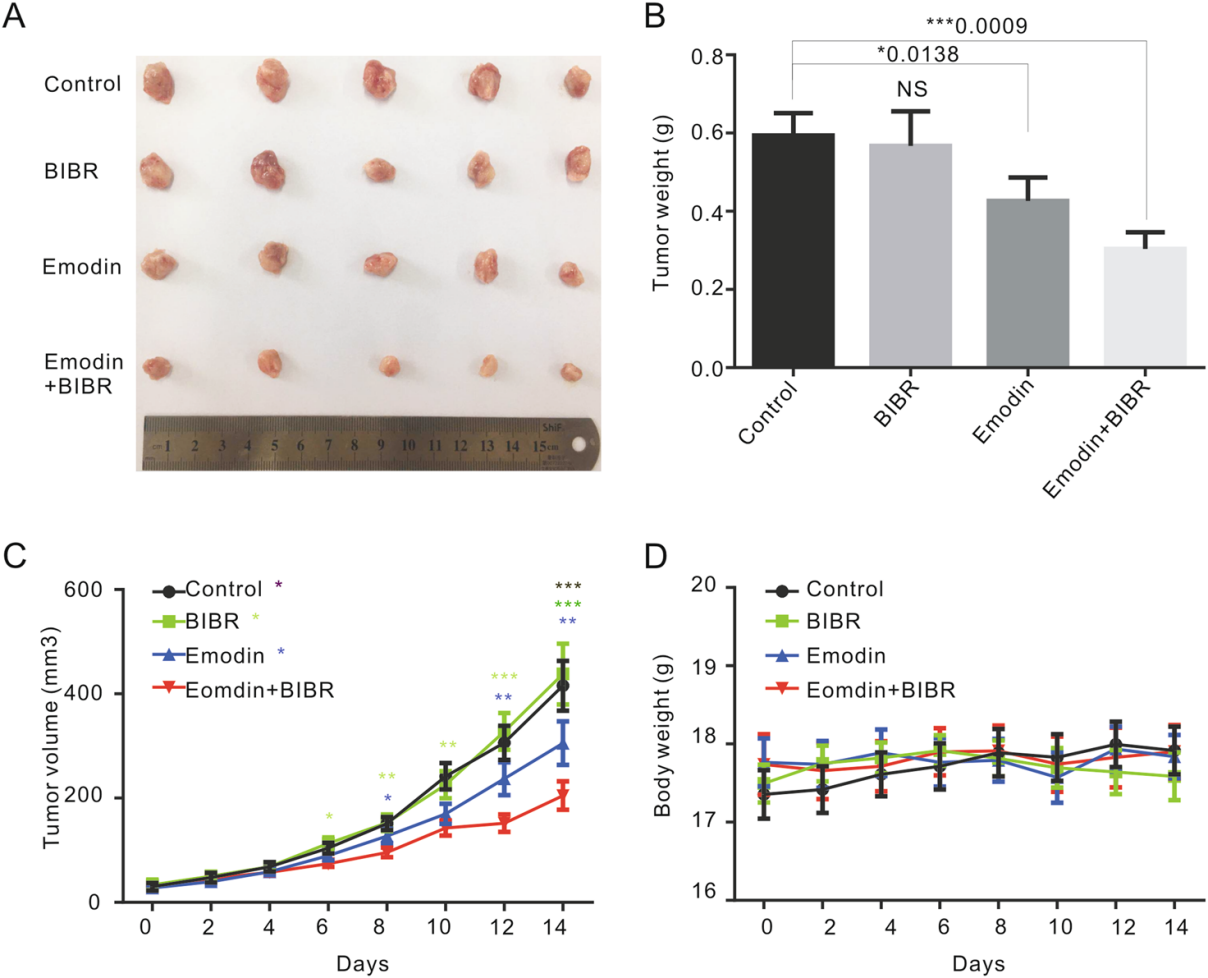
The combination treatment of emodin and BIBR1532 can significantly inhibit tumor growth. **a** The breast cancer of different drugs treatment groups. **b** Tumor weight. **c** Tumor growth curves. The colored asterisks represent comparison of each group with the combination treatment of emodin and BIBR1532. **d** Body weight of mice were measured every 2 days. Data are shown as mean ± SD. *n* = 18. Values are represented as mean ± SD

To further investigate the combined effect of emodin and BIBR1532 treatment on tumor growth in vivo, the above compounds were applied to 4T1 cell xenograft breast cancer mice either simultaneously or separately. A total of 20 tumor model mice were randomly divided into 4 groups, with 5 mice per group; these groups were a control group administered 0.5% CMC-Na by oral gavage, an emodin treatment group receiving emodin was dissolved in 0.5% sodium carboxymethyl cellulose (CMC-Na) by oral gavage (25 mg/kg/d vs 20 mg/kg/d, *n* = 18), a BIBR1532 treatment group administered BIBR1532 by intratumoral injection (10 mg/kg/d) and an emodin plus BIBR1532 treatment group. All treatments were performed for 14 days. The numbers of injected tumor cells were the same in different groups, and there were only modest changes in the first 6 days after treatment. However, the tumor weight analysis data showed that emodin but not BIBR1532 treatment slightly decreased tumor size (Fig. 7a, b), and the average tumor weight decreased 30% after two weeks of emodin treatment. Moreover, combined treatment with emodin and BIBR1532 significantly inhibited tumor growth compared to BIBR or emodin single treatment (Fig. 7b, c) without obvious changes in body weight (Fig. 7d). Therefore, these results confirmed that increased telomerase activity may play a key role during emodin-induced telomere dysfunction recovery and that the inhibition of telomerase upon emodin treatment leads to permanent telomere damage and tumor growth inhibition, indicating that the combination of emodin and BIBR1532 represents a potential oncotherapy for telomerase-positive tumors.

## Discussion

This study provides valuable insight into the potential mechanism whereby emodin inhibits cancer cell growth. We observed that emodin exposure facilitated the formation of G4s in the telomeric region and induced telomere dysfunction, which led to cellular apoptosis and senescence. However, subsequent telomerase activation restored the damaged telomeres and rescued telomere dysfunction. We report for the first time that combined treatment with emodin and telomerase inhibitors induces permanent telomere DNA damage and significantly inhibits tumor growth. Furthermore, our data suggested that the combined use of telomerase inhibitors and other G4 chemical stabilizers may be applied for telomerase-positive cancer therapy.

During emodin treatment, we observed that emodin-induced significant cellular apoptosis in addition to senescence. It has been established that both apoptosis and senescence can function under cellular stress to arrest proliferation^47^. However, apoptosis and senescence are two independent cellular dysfunction response mechanisms that impair cellular function, and they do not occur at the same time^48^. Apoptosis is a programmed cell suicide process that might result from DNA damage (as well as telomere DNA damage) in the absence of DNA repair^49^. Unlike apoptosis, senescence is an active cytostatic program that occurs in response to proliferative stress and toxicity and is increased in aging cells or tissues^50^. Based on the underlying mechanism, cellular senescence can be divided into two subgroups: replicative senescence and premature senescence. Replicative senescence is most likely triggered by telomere erosion, which can be rescued by the restoration of telomerase. In contrast, premature senescence tends to be induced by severe DNA damage, overexpression of oncogenes or loss of tumor suppressors^51,52^. Although the stimuli involved are different, the gene expression profile shows high identity between these two groups^53^. Here, we observed significantly increased telomere DNA damage and disturbance upon emodin treatment, but it will be difficult to clarify whether apoptosis, replicative senescence or premature senescence is the dominant pathway inducing cellular proliferation arrest.

The stabilization of G4s at telomeres has been reported to lead to telomere dysfunction^54^ by disturbing telomere replication^21,22^. In addition, emodin exposure increases the formation of G4s in cells. Thus, the significantly increased MTS, SFE, and TIF signals may indicate a telomeric effect of accumulated G4s. However, emodin has been reported to elicit DNA DSBs by inhibiting Topoisomerase^26,55^, an enzyme that also plays an essential role in telomeric DNA protection^56^. Thus, we cannot rule out the possibility that the observed telomeric DNA damage is due to the disturbance of topoisomerase II upon emodin treatment.

Damaged telomeres can be recognized and elongated by telomerase, which requires ATM/ATR kinase for assembly and recruitment^13,57^. More recently, telomerase activity has been reported to be increased by either ATR or ATM signals and then adds de novo telomeric DNA to damaged sites elsewhere in the genome for repair purposes^58^. The possible reasons for the increase in telomerase activity upon emodin treatment include ATR activation following G4-induced replication fork stalling and ATM activation resulting from emodin-induced double-strand DNA breaks. In turn, the increased telomerase activity overrides both single-strand breaks (ATR) and double strands breaks (ATM) with time. The recovery of DNA damage, especially at the later times after emodin exposure, may have resulted from increased telomerase repair activity, which could only be identified in telomerase-positive cells. Thus, a higher concentration of emodin is required to halt the proliferation of cells in which telomere damage is rescued.

Emodin has not been widely used as an oncotherapeutic agent due to its poor bioavailability, high cytotoxicity at high concentrations and the occurrence of drug resistance. Thus, the search for an approach that could synergistically increase its bioactivity at lower concentrations is very important. Telomerase inhibitors can inhibit telomerase activity and impede telomere elongation. Treatment with the telomerase inhibitor BIBR1532 greatly suppresses emodin-induced telomerase upregulation, emphasizing that emodin triggers telomere dysfunction and subsequent cellular apoptosis and senescence in telomerase-positive cancer cells, indicating this combination as a potential antitumor agent. This study contributes to further research on the future clinical application of emodin.

## Supplementary information

Combined treatment with emodin and a telomerase inhibitor induces significant telomere damage/dysfunction and cell death
