# Supplementary material for: Combined treatment with emodin and a telomerase inhibitor induces significant telomere damage/dysfunction and cell death
Source: Cell Death Dis. 2019 Jul 11;10(7):527. doi: 10.1038/s41419-019-1768-x (PMC6624283; doi:10.1038/s41419-019-1768-x)
Supplement: Supplementary file 1 — Combined treatment with emodin and a telomerase inhibitor induces significant telomere damage/dysfunction and cell death [file 41419_2019_1768_MOESM1_ESM.pdf]

Figure S1

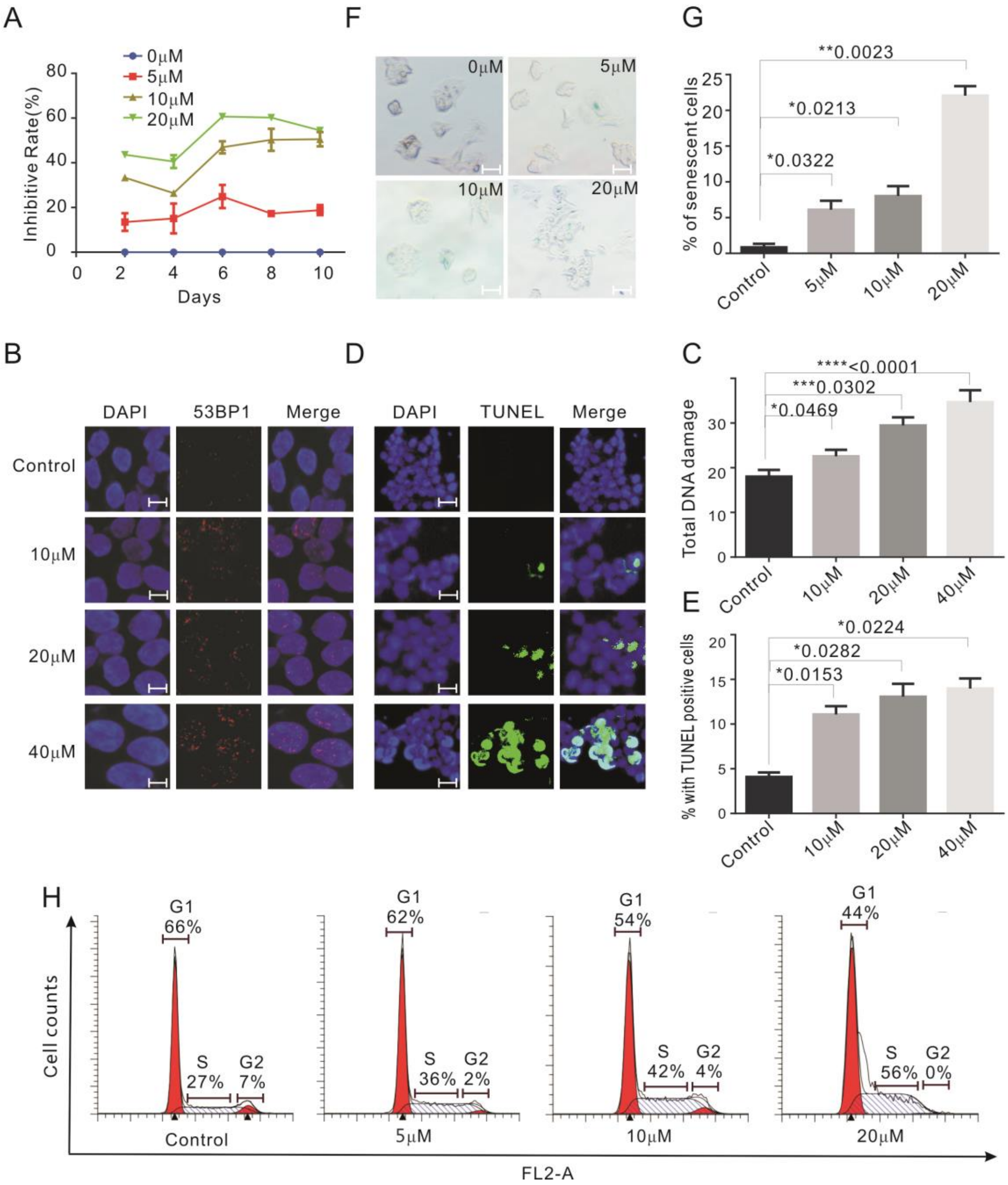
A
0μM
5μM
10μM
20μM
Inhibitive Rate(%)
Days
F
0μM
5μM
10μM
20μM
G
**0.0023
*0.0213
*0.0322
% of senescent cells
Control
5μM
10μM
20μM
B
DAPI
53BP1
Merge
Control
10μM
20μM
40μM
D
DAPI
TUNEL
Merge
C
****<0.0001
***0.0302
*0.0469
Total DNA damage
Control
10μM
20μM
40μM
E
*0.0224
*0.0282
*0.0153
% with TUNEL positive cells
Control
10μM
20μM
40μM
H
Cell counts
G1 66%
S 27%
G2 7%
Control
G1 62%
S 36%
G2 2%
5μM
G1 54%
S 42%
G2 4%
10μM
G1 44%
S 56%
G2 0%
20μM
FL2-A

Figure S2

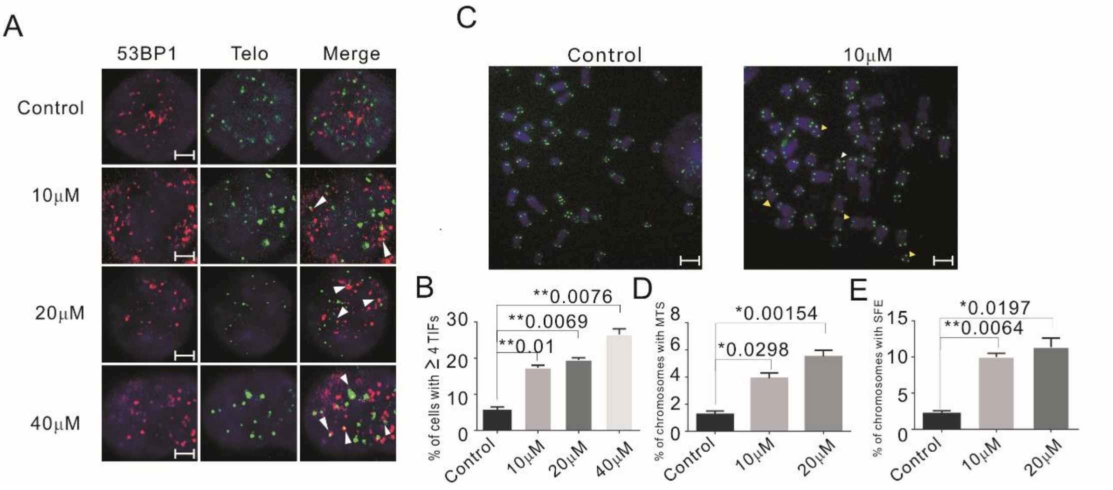

Figure S3

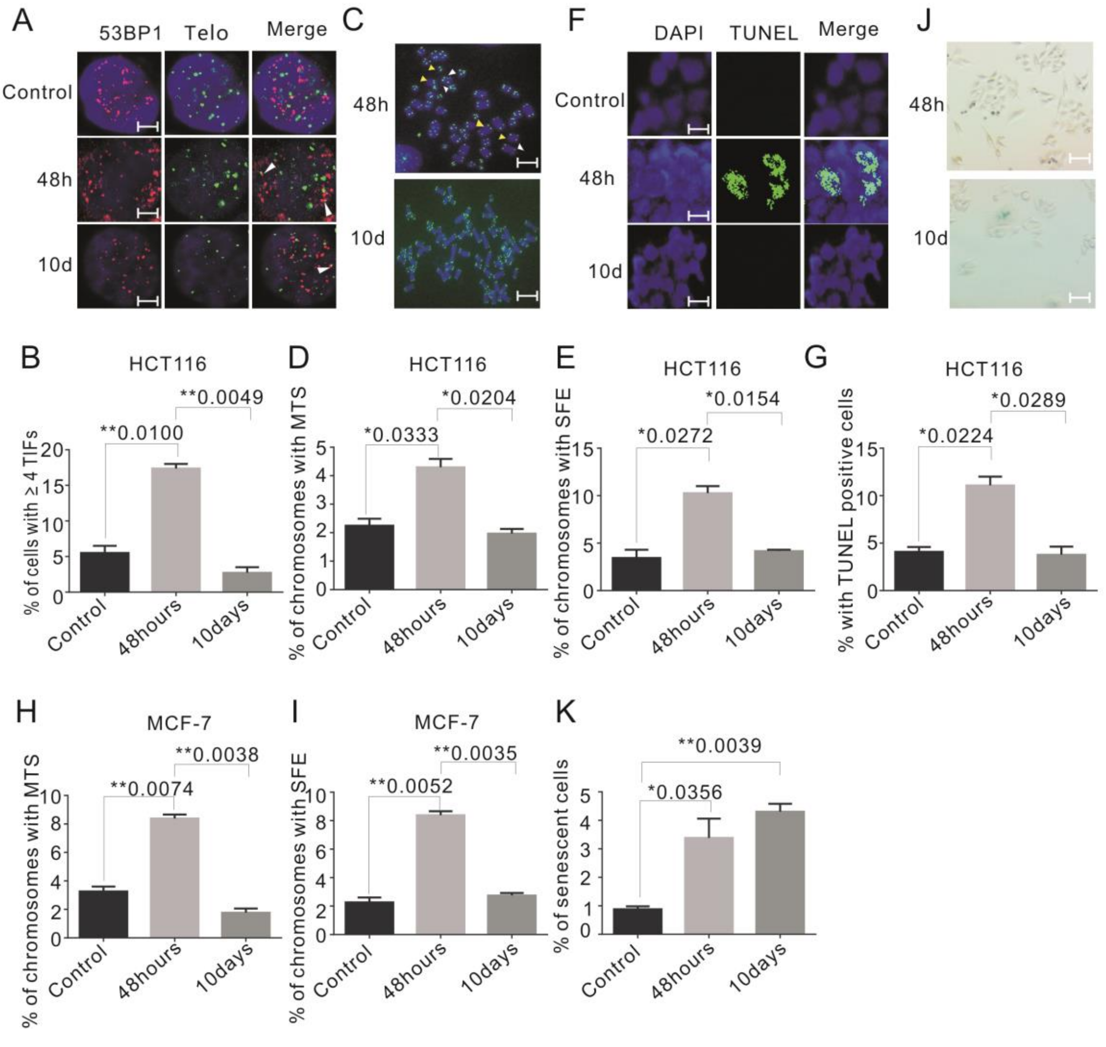

Figure S4

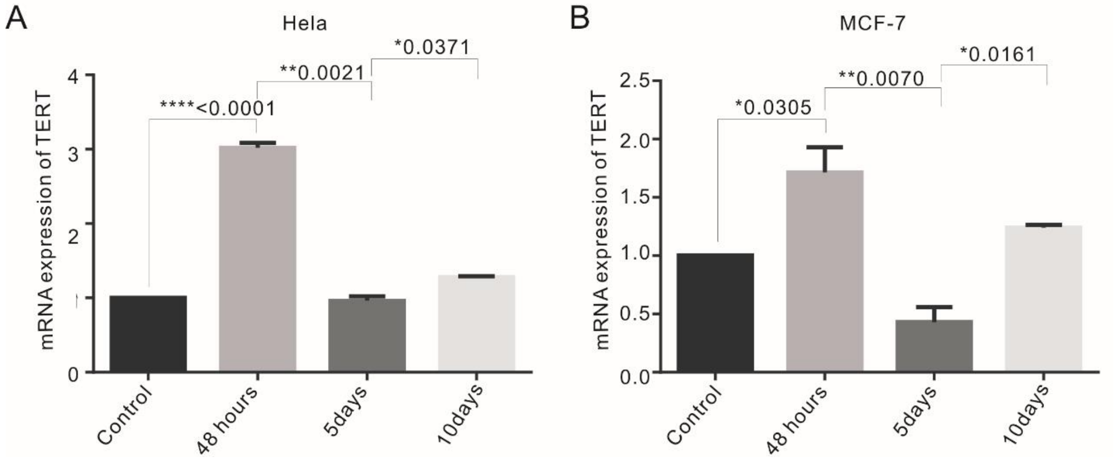
A
Hela
mRNA expression of TERT
4
3
2
1
0
****<0.0001
**0.0021
*0.0371
Control
48 hours
5days
10days
B
MCF-7
mRNA expression of TERT
2.5
2.0
1.5
1.0
0.5
0.0
*0.0305
**0.0070
*0.0161
Control
48 hours
5days
10days

Figure S5

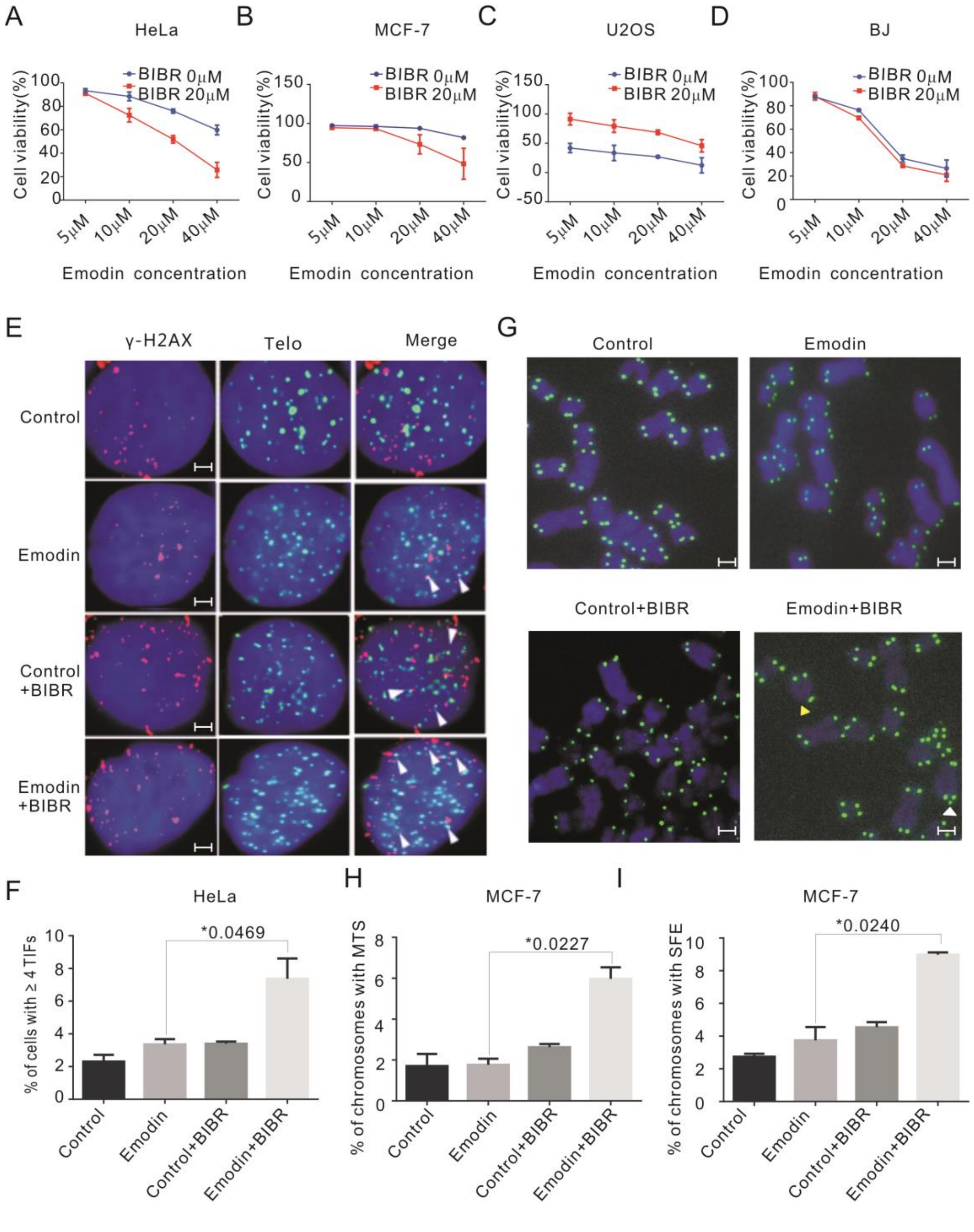
A
HeLa
Cell viability(%)
BIBR 0μM
BIBR 20μM
Emodin concentration
B
MCF-7
C
U2OS
D
BJ
5μM
10μM
20μM
40μM
E
γ-H2AX
Telo
Merge
Control
Emodin
Control +BIBR
Emodin +BIBR
G
Control
Emodin
Control+BIBR
Emodin+BIBR
F
HeLa
% of cells with ≥ 4 TIFs
*0.0469
H
MCF-7
% of chromosomes with MTS
*0.0227
I
MCF-7
% of chromosomes with SFE
*0.0240
Control
Emodin
Control+BIBR
Emodin+BIBR

Figure legends

Figure S1. Emodin causes DNA damage, proliferation inhibition, apoptosis and senescence in HCT116 cells. A. Inhibitive rate of HCT116 cells treated with DMSO and 5 μM, 10 μM and 20 μM emodin for 10 d. **B.** Representative images showing the detection of 53BP1 for the control (DMSO) and emodin treated HCT116 cells (10 μM, 20 μM and 40 μM for 48 h).53BP1 (red), nuclei (blue). Scale bars are 10 μm. **C.** The graph of the number of DNA-damage foci for B. **D.** Representative images showing apoptotic cells detected by TUNEL. TUNEL positive cells labeled as green. Cells were treated with with DMSO, 10 μM, 20 μM and 40 μM emodin for 48 h. Scale bars are 20 μm. **E.** The graph of D. **F.** Cells were treated with DMSO and 5 μM, 10 μM and 20 μM emodin in for 48 h, then sensecense cells were detected by SA-β-gal staining. SA-β-gal positive cells were stained in blue. Scale bars are 20 μm. **G.** The percentage of senescent cells with different treatment. **H.** Cell cycle distribution was determined with fluorescence-activated cell–sorting (FACS) analysis. HeLa cells were treated with DMSO and 5 μM, 10 μM and 20 μM emodin in for 48 h. Data are shown as mean ± SD. n=3.

Figure S2. Emodin induces telomere specific DNA damage and dysfunction by acute exposure in HCT116 cells. A. Immunolocalization of 53BP1 (red) and FISH of telomere (green) in HCT116 cells grown with or without emodin for 48 h. White arrows indicate TIFs (sites of 53BP1 with telomeres). Scale bars are 2 μm. **B.** The percentage of cells with ≥ 4 TIFs was determined for at least 50 cells in each experiment. **C.** Telomere FISH with HCT116 cells treated with indicate amount of emodin for 48 h. White arrows indicate MTS and yellow arrows indicate SFE. Scale bars are 5 μm. **D.** & **E.** Percentage of chromosomes with MTS and SFE. More than 1000 chromosomes were analyzed in each experiment. Data are shown as mean ± SD. n=3.

Figure S3. Emodin causes telomere dysfunction that can be repaired after chronic treatment in HCT116 and MCF-7 cells. A. Representative images showing the TIFs in control (DMSO for 10 d) and 10 μM emodin treated HCT116 cells (48 h and 10 d) 53BP1 (red), telomere (green). Nuclei were stained with DAPI (blue). White arrows in merged images indicate TIFs. Scale bars are 2 μm. **B.** The graph showing the percentage of TIFs with different treatment. **C.** Representative images of metaphase telomere FISH. HCT116 cells were treated with emodin in 10 μM for 48 h and 10 d. White arrows indicate MTS and yellow arrows indicate SFE. Scale bars are 5 μm. **D.** & **E.** Percentage of chromosomes with MTS and SFE for different treatment. **F.** Representative images of TUNEL positive Percentage of apoptosis cells were detected by TUNEL with indicated condition. Scale bars are 20 μm. **G.** Graph showing the percentage of TUNEL positive cells after emodin treatment. **H.** & **I.** P Percentage of chromosomes with MTS and SFE for different treatment in MCF-7 cells. **J.** HeLa cells were treated with emodin in 10 μM for 48 h and 10 d, then sensecense cells were detected by SA-β-gal staining. SA-β-gal positive cells were stained in blue. Scale bars are 20 μm. **K.** The percentage of senescent cells with different treatment. Data are shown as mean ± SD. n=3.

Figure S4. Telomerase activity was elevated in the first few days upon emodin treatment and recovered late on. A. & **B.** Relative expression of hTERT mRNA transcripts in HeLa and MCF-7 cells. HeLa treated in 5 μM for 48 h, 5 d and 10 d. MCF-7 treated in 10 μM for 48 h, 5 d and 10 d. Data are shown as mean ± SD. n=3.

Figure S5. The repair of telomere dysfunction does not occur when telomerase activity is inhibited in telomerase positive cancer cells. A. &. **B.** Cell viability dramatically decreased with the combined treatement of different concentrations (5 μM, 10 μM, 20 μM and 40 μM) emodin and 20 μM telomerase inhibitor BIBR1532 in HeLa and MCF-7 cells. **C.** Cell viability was not affected by telomerase inhibitor BIBR1532 in telomerase negative cancer cells U2OS. **D.** Cell viability was not affected by telomerase inhibitor BIBR1532 in telomerase-null normal fibroblasts BJ. **E.** Immunolocalization of γ-H_2AX (red) and FISH of telomere (green) in HeLa cells. Control (DMSO for 10 d), emodin group (5 μM for 10 d), BIBR group (DMSO for 48 h, then 20 μM BIBR1532 was added for additional 8 d), emodin and BIBR group (emodin 5 μM for 48 h, then 20 μM BIBR1532 was added for additional 8 d). White arrows indicate TIFs (sites of 53BP1 with telomeres). Scale bars are 2 μm. **F.** The percentage of cells with ≥4 TIFs was determined for at least 50 cells in each experiment. **G.** Representative images of metaphase telomere FISH. Control (DMSO for 10d), emodin group (10 μM for 10 d), BIBR group (DMSO for 48 h, then 20 μM BIBR1532 was added for additional 8 d), emodin and BIBR group (emodin 10 μM for 48 h, then 20 μM BIBR1532 was added for additional 8 d). White arrows indicate MTS and yellow arrows indicate SFE. Scale bars are 3 μm. **H.** & **I.** Percentage of chromosomes with MTS and SFE with different treatment. Data are shown as mean ± SD. n=3.
